# Temperature-Induced Restructuring of Mycolic Acid Bilayers Modeling the *Mycobacterium tuberculosis* Outer Membrane: A Molecular Dynamics Study

**DOI:** 10.3390/molecules29030696

**Published:** 2024-02-02

**Authors:** Alexander V. Vasyankin, Sergey V. Panteleev, Ilya S. Steshin, Ekaterina A. Shirokova, Alexey V. Rozhkov, Grigory D. Livshits, Eugene V. Radchenko, Stanislav K. Ignatov, Vladimir A. Palyulin

**Affiliations:** 1 Department of Chemistry, Lobachevsky State University of Nizhny Novgorod, Nizhny Novgorod 603022, Russia; alexandrvasyankin@gmail.com (A.V.V.); pasv1984@gmail.com (S.V.P.); ilya.steshin@icloud.com (I.S.S.); ekashirokova@gmail.com (E.A.S.); rozhkov@chem.unn.ru (A.V.R.); grigory.livshits@gmail.com (G.D.L.); genie@qsar.chem.msu.ru (E.V.R.); 2 Department of Chemistry, Lomonosov Moscow State University, Moscow 119991, Russia

**Keywords:** tuberculosis, mycobacterium, cell wall, outer membrane, temperature induced conformational changes, diffusion constants, lipid ordering, activation energies, molecular dynamics

## Abstract

The emergence of new drug-resistant strains of the tuberculosis pathogen *Mycobacterium tuberculosis* (Mtb) is a new challenge for modern medicine. Its resistance capacity is closely related to the properties of the outer membrane of the Mtb cell wall, which is a bilayer membrane formed by mycolic acids (MAs) and their derivatives. To date, the molecular mechanisms of the response of the Mtb outer membrane to external factors and, in particular, elevated temperatures have not been sufficiently studied. In this work, we consider the temperature-induced changes in the structure, ordering, and molecular mobility of bilayer MA membranes of various chemical and conformational compositions. Using all-atom long-term molecular dynamics simulations of various MA membranes, we report the kinetic parameters of temperature-dependent changes in the MA self-diffusion coefficients and conformational compositions, including the apparent activation energies of these processes, as well as the characteristic times of ordering changes and the features of phase transitions occurring over a wide range of elevated temperatures. Understanding these effects could be useful for the prevention of drug resistance and the development of membrane-targeting pharmaceuticals, as well as in the design of membrane-based materials.

## 1. Introduction

Tuberculosis (TB) is a severe disease caused by the *Mycobacterium tuberculosis* (Mtb) pathogenic microorganism. Today, it is one of the most threatening infections worldwide, characterized by high latent morbidity as well as high mortality in its acute form [1,2,3,4]. Of particular danger is the emergence of Mtb strains with multi-drug resistance (MDR) and extensive drug resistance (XDR) against known antibiotics. This is currently seen as a critical new challenge for modern medicine that requires the urgent development of new antimicrobial agents to combat Mtb. However, in the process of their evolution, mycobacteria have developed an extremely stable protective wall that hinders the penetration of antibiotics into the cell and protects it against the action of the host immune system and environmental conditions. The outer protective layer of the Mtb cell wall is the exceptionally tough and dense shell that is up to 8 nm thick and called the outer membrane (OM) [5]. It is a bilayer membrane consisting mainly of mycolic acids (MAs) and their derivatives. Most of MA molecules in the more rigid inner leaflet are covalently linked to the underlying layers of arabinogalactan and peptidoglycan, while the outer leaflet comprises free MAs, mycolate esters of trehalose and other alcohols, glycopeptidolipids, and other lipid components, with a total MA content several times lower than in the inner leaflet [6,7,8].

**Figure 1:**
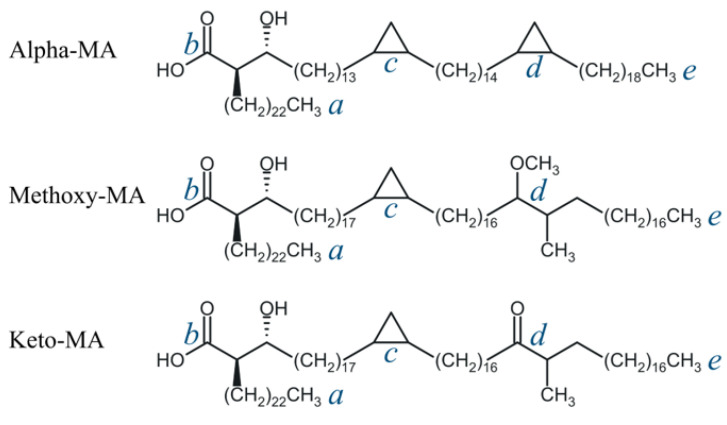
Structures of the AMA, MMA, and KMA mycolic acid molecules considered in this study. The letters *a*–*e* mark the key elements of the structure: *a*—end of the α-chain; *b*—carboxyl group, *c*—proximal functional group, *d*—distal functional group, *e*—end of the long hydrocarbon (meromycolate) chain.

MAs are 2-alkyl-3-hydroxy long-chain fatty acids that have slightly different chain lengths and additional chemical groups both across and within species [9,10,11,12,13]. In the Mtb outer wall, the most abundant components are α-mycolic acid (AMA), ketomycolic acid (KMA), and methoxymycolic acid (MMA) (Figure 1). All of them are β-hydroxy acids with linear chains of 60–90 carbon atoms [14]. These molecules have a linear aliphatic chain of about 23 carbon atoms (fragment *a*–*b* in Figure 1) at the α-position with respect to the COOH group (*b*) and a hydroxy group at the β-position, corresponding to the (2R,3R) stereochemistry. Two more groups are found at positions *c* and *d* inside a long hydrocarbon (meromycolate) chain. AMA has *cis*-cyclopropane rings inserted in both the proximal (*c*) and the distal (*d*) positions with respect to the carboxyl group. It is usually the most abundant type of MA in Mtb, comprising from 50% to 70% of the total MA content [15,16,17]. MMA and KMA, collectively comprising from 30% to 50% of the total MA content, have either *cis*- or α-methyl-*trans*-cyclopropane rings inserted in the proximal (*c*) position, and, respectively, methoxy and methyl or keto and methyl substituents in the distal (*d*) position [12,14].

The structure and properties of mycolate membranes are currently of great interest due to their protective role for mycobacteria. In particular, it was found [18,19] that MAs enhance Mtb resistance against chemical damage and dehydration, as well as reduce the efficiency of hydrophilic antibiotics and biocides. MAs also enable the bacterium to grow within macrophages, effectively concealing it from the host immune system [16].

It is known [20,21,22,23] that bacteria are capable of producing cell wall changes in response to changes in the environment and to temperature fluctuations in particular, which represents a strategy developed to adapt to environmental conditions. It is highly likely that one of the mechanisms of such adaptation in mycobacteria is based on adjustments in the ratio of different mycolic acids and their structural organization. The temperature dependence of the structure and mechanical properties of mycolic membranes is poorly investigated, although there are quite a few experimental studies of such dependencies for lipid membranes [24,25,26,27,28,29,30,31,32,33,34,35].

The effect of elevated temperatures on mycolic membranes was studied by detecting the high-temperature phase transitions in the ‘bulk’ purified cell walls of various mycobacteria using the differential scanning calorimetry (DSC) method [36]. The transition temperature depended on the MA chain length. In most samples, two transitions were observed in the regions of 20–40 °C and 60–70 °C. Moreover, the transition temperature depended on the biological species, and in the case of *M. terrae*, the high-temperature transition was observed in the region of 90–95 °C.

In [37], artificially synthesized in vitro lipid membranes containing MAs and their various glyco-derivatives were studied by fluorescence spectroscopy, FT-IR spectroscopy, and atomic force microscopy. Different components of *M. smegmatis* (Msm) envelopes extracted from living bacteria were used for synthesis, and the synthesized membranes differed in lipid composition. It was found that the model membranes simulating the outer Msm membrane were highly mobile and dynamic. A fluorescence spectroscopy study using Laurdan dye demonstrated structural changes in all membranes in the 40–50 °C region. For other dyes, the changes were less pronounced. FT-IR spectroscopy showed changes in the membrane structure in the regions of 20–30 °C and 30–40 °C depending on the sample. Atomic force microscopy showed the formation of a domain structure on the membrane surface.

When studying bulk solid AMA, KMA, and MMA by DSC, it was found that their melting temperatures are in the range of 50–70 °C, and the corresponding melting enthalpies are in the range of 21–30 kcal/mol. Thus, the melting temperatures of MA are close to the melting temperatures of lighter fatty acids (myristic and palmitic acids), but their melting enthalpies are about twice as high (10.5–13 kcal/mol for myristic and palmitic acids) [38]. It can be seen that the melting temperature of solid MAs is close to the high-temperature phase transitions observed in membranes. However, in most works, the low-temperature transitions in the range of 20–40 °C are attributed to transitions from the gel to liquid crystalline state. It is possible that these observed transitions are not associated with mycolic acids but rather with more mobile lipids of the outer leaflet of the membrane.

In [39], the heat capacity of the organized lipid domains of the cell wall isolated from *M. chelonae* was studied by the DSC method. Phase transitions were found at temperatures of about 30, 50, and 60 °C. This study was further developed in [36], where different *Mycobacterium* and *Corynebacterium* species were considered. In particular, phase transitions were found for *M. tuberculosis* at temperatures of about 30 and 60 °C (for the cell wall), as well as of about 40 °C (for mycolic acid methyl ester).

The literature analysis indicates that the processes occurring in the mycolic acid membranes under increasing temperatures are not sufficiently studied. In particular, there is practically no information on the effect of temperature on the molecular ordering in membranes (including the nature and timing of changes in this ordering) or on the changes in their conformational composition. A detailed analysis of such information could significantly facilitate the understanding of the relationships between the temperature and properties of the mycobacterial cell wall, as well as the processes underlying the biochemical regulation of its properties. One approach that provides insight into these processes at the molecular level is based on the molecular dynamics method. Although many cooperative effects can be investigated by coarse-grained MD simulations, the details of the structural organization are more accurately described by the full-atom force fields.

Only a few works aiming to study mycolic membranes using MD methods with full-atom force fields have been published. In a series of works by Hong and Hopfinger [40,41], single-layer membranes modeling the Mtb membrane were constructed and the diffusion of drug compounds through them was studied. Among the components of the model membranes were mycolyl arabinan complexes and MA-like molecules that mimic the inner leaflet of the outer Mtb membrane. Based on 90–210 ps MD simulations using simple molecular mechanics force fields, it was concluded that the MA molecules formed dense ordered layers, which confirmed the structure of the Mtb outer membrane. Later, in a combined experimental and theoretical study [42], the MD modeling of a trehalose 6,6′-dimycolate (TDM) bilayer simulating the outer leaflet of the Mtb outer membrane was carried out. The membrane contained 60 molecules of TDM. NPT MD (*T* = 300 K) was performed with a force field based on GLYCAM-06 and GAFF, and the trajectory length was 40 ns. The molecular structure of glycolipids in the membrane, including the trehalose conformations, and the density profiles were analyzed. The bilayer thickness and area per lipid were determined as 4.2 nm and 103.9 Å^2^, respectively. The MD modeling was also performed in a combined study [43] to investigate the effect of rifabutin on the inner and outer membrane properties of Mtb, as well as its diffusion across these membranes. The outer membrane bilayer consisted of 800 lipids of different types. The NPT molecular dynamics of 1 µs duration with GAFF and a Lipid-14-based force field at *T* = 300 K was studied. In addition to the effects of rifabutin on membrane properties, the estimates of the self-diffusion coefficients for different types of lipids, including pure mycolic acids (0.09 ± 0.01 µm^2^/s), and characterizations of the membrane domain structure were obtained. Recently, in our study [44], the conformational composition and the dynamics of its changes over time, density profiles, and other structural characteristics of the AMA/KMA/MMA bilayer membranes of different initial structures were investigated. NPT MD calculations were performed using the CHARMM36 force field, with a trajectory length of up to 1.2 µs at *T* = 300 K.

As follows from this overview, no MD studies of the effects of temperature on the outer Mtb membrane have been published. In this work, we investigate the changes in the structure of the symmetric bilayer mycolic acid membrane, serving as a model of the outer Mtb membrane, under the influence of elevated temperatures. Although the molecules of the inner leaflet in the natural mycobacterial membrane are covalently bound to the underlying glycoproteins and the outer layer is significantly enriched in glycolipids, we consider the symmetric membranes of free MA molecules as a simple model that can reproduce important properties of the original membranes, particularly the packing features of long molecules and the temperature-induced changes in their conformations and mobility. Using the classical all-atom molecular dynamics (MD) method, we study the various quantitative features of ordering in these membranes that allow us to analyze the effect of temperature on the membrane structure, including phase transitions. As parameters that characterize the ordering and mobility of membrane molecules, we consider changes in the membrane conformational composition and the rate of these changes; the self-diffusion coefficients of membrane molecules and their temperature dependence; and the temperature dependence of the orientational ordering of the molecules. It is expected that understanding these effects could be useful for the prevention of drug resistance and the analysis of factors influencing the heat resistance of mycobacteria, as well as the development of membrane-targeting pharmaceuticals [45,46,47,48] and the design of membrane-based materials.

## 2. Results and Discussion

### 2.1. Model Systems and Conditions

In keeping with the approach employed in our previous works [44,49] to study the structures of mycolate-based outer Mtb membranes and their free energy profiles regarding the transverse diffusion of drug molecules, specially constructed model membranes based on symmetrical mycolic acid bilayers with different component compositions and thicknesses were considered in the analysis. We believe that the choice of symmetrical systems consisting of free MA molecules is justified because they represent rather simple prototypical models that allow one to significantly reduce the number of variable parameters and thus the uncertainty and complexity of modeling. In fact, the membrane model is determined by only three factors: (1) the MA component composition, i.e., the ratios and counts of the AMA, KMA, and MMA molecules; (2) the initial conformational composition, which determines the density, thickness, and free volume of membranes; and (3) the ionization state of MA molecules. As we will see, even such a simple model supports fairly wide variations in the membrane properties and provides valuable results. This model is certainly limited because it does not reflect (1) the covalent linkages between the inner leaflet MAs and the underlying arabinogalactan and peptidoglycan layers, and (2) a significant amount of relatively smaller and more mobile lipids (including mycolate esters) in the outer leaflet. Thus, the model is likely to somewhat overestimate the inner leaflet fluidity and underestimate the outer leaflet fluidity compared to the natural membranes. However, the mycolate bilayer serves as a strong “framework” that determines the OM’s protective properties, and the MA packing and voids in it largely control the diffusion [49]. The inner leaflet’s barrier role is believed to dominate, and the overall model bias in the analysis of diffusion and temperature effects should not be critical. At the same time, the symmetrical unbound bilayer greatly simplifies the modeling by avoiding the need to properly represent a quasi-solid support layer and account for significantly higher molecular diversity in the system. In the future, this model can be easily refined and expanded to include these new features while building upon the previously identified patterns of membrane property dependence on other parameters.

In the course of this study, three bilayer membrane types were adopted from the previous study [44]. The first one, designated as AMA_W, was initially constructed from the AMA molecules in their W conformations (see [44] for the conformation designations), which undergo significant conformational restructuring. The second pure AMA bilayer membrane, designated as AMA_eU and built with the initial eU conformations, has the greatest density and thickness. The third was the mixed membrane constructed from three components: AMA (50%), KMA (25%), and MMA (25%), with the AMA molecules initially having eU conformations. This composition is close to the natural composition of the Mtb outer membrane and is designated as Mix50_eU. In order to estimate the influence of the environmental acidity, its partially ionized form Mix50_eU_ion was additionally considered. The chemical and conformational compositions of the studied membranes, as well as their structure and density characteristics, are presented in detail in Section 3.1.

In contrast to phospholipid-based cytoplasmic membranes, mycolate membranes have significantly higher viscosity. Taking this into account, in the present study, we focus on a relatively higher temperature range (*T* = 310–370 K or 37–97 °C) that is relevant from several points of view. First, it allows one to explore the temperature limits at which the Mtb membrane remains in certain structural states. Second, this makes it possible to find out whether the experimentally observed changes in the membrane fluidity are associated with the properties of the MA layers themselves or with more mobile lipids in the outer OM leaflet. Third, there are heat-resistant and thermophilic mycobacteria strains and species (for example, *M. terrae*) that have membrane phase transitions observed in the temperature range of 360–370 K. In addition, in all the membranes considered in this study, no significant structural changes were observed at lower temperatures (see Section 2.4).

### 2.2. Temperature-Induced Conformational Changes in the MA Membranes and Their Activation Energies

**Figure 2:**
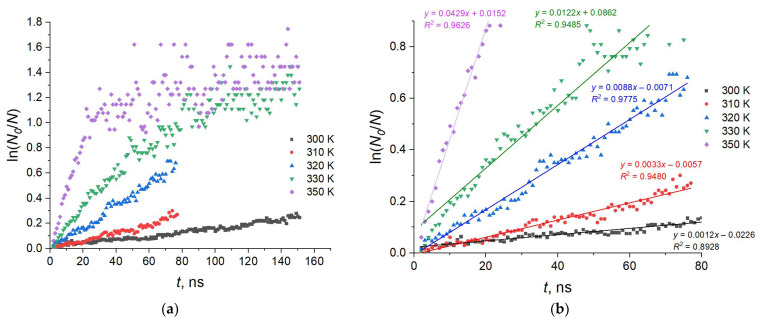
Decrease in the W conformation counts in the AMA_W membrane over time at different temperatures: (**a**) between 0–160 ns; (**b**) between 0–80 ns.

**Figure 3:**
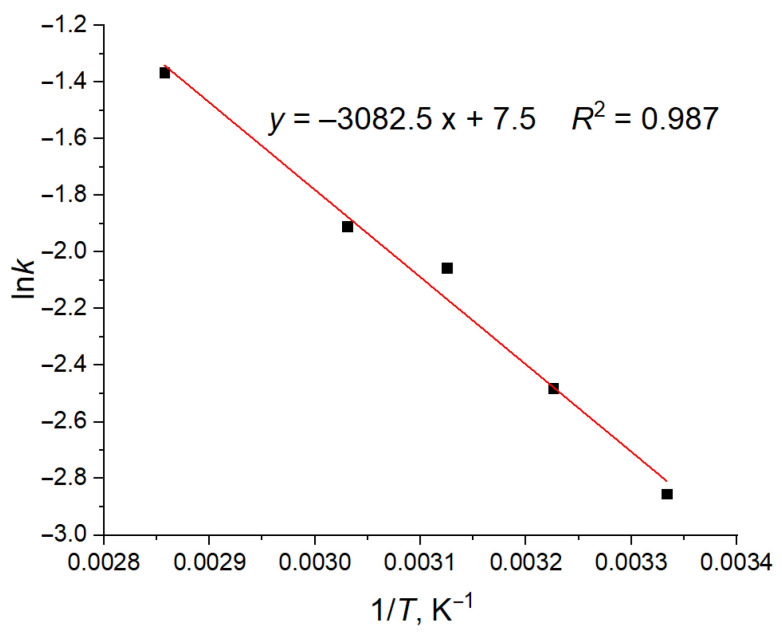
Dependence of the logarithm of the rate constant of W conformation decay in the AMA_W membrane on the inverse temperature. Individual data points (black squares) and linear regression model (red line) are shown.

One of the characteristics most sensitive to temperature is the conformational composition of the membranes. It was shown earlier [44] that even at 300 K, the conformational composition changes markedly during the first 500–600 ns of the MD trajectory. These changes in the case of the AMA_W membrane represented the transformation of the basic initial W conformation into other conformation types that obeyed the first-order reaction kinetics. At the increased temperatures, this feature was retained. Figure 2a shows the temperature dependence of the ln(*N*_0_/*N*) kinetic curves (where *N*_0_ and *N* are the initial and current counts of the W conformation molecules in the AMA_W membrane, respectively) in the time range of 100–160 ns. It can be seen that at higher temperatures, the kinetic curves reach a plateau by 30–100 ns. The initial sections of the curves (in the time range of 0–50 ns) are shown in Figure 2b. It can be seen that all kinetic curves are close to linear, confirming the first kinetic order of the W conformation “decay” process. Its apparent decay rate constant *k* is expected to follow the Arrhenius equation:

*k* = *k*_0_ exp(−*E*_a_/*RT*), (1)

where *k*_0_ is the preexponential factor; *R* is the universal gas constant; *T* is the absolute temperature, and *E*_a_ is the apparent activation energy of the process. Indeed, the dependence of the logarithm of the rate constant *k* on the inverse temperature is also close to linear (Figure 3), allowing us to define the apparent activation energy of conformational changes *E*_a_ as 6.13 kcal/mol. Obviously, the physical meaning of this value reflects the activation energy of the diffusion of individual MA molecule chains in the membrane. The changes in the conformation counts and their apparent rate constants *k* are determined by the mobility of individual fragments of MA molecules (for example, bending or elongation of their hydrocarbon chains), which also determines the possibility of the molecules diffusing as a whole and, thus, the values of the diffusion coefficient. Consequently, it can be compared to the diffusion activation energies in various membranes. Indeed, the conformation-based results are consistent with the experimental activation energies of the lateral diffusion of phospholipid molecules in the “liquid” (L_d_) phase of phospholipid membranes, which were measured as 6–10 kcal/mol for sphingomyelin (SM), palmitoyloleoylphosphatidylcholine (POPC), dimyristoylphosphatidylcholine (DMPC), dioleoylphosphatidylcholine (DOPC), and dipalmitoylglycerophosphocholine (DPPC) [50,51,52,53].

In contrast to the W conformation, the eU conformation in mycolate membranes is significantly more stable and the number of observed conformational transformations is small, precluding us from determining their activation energies. Therefore, to analyze the temperature effects on the MA molecule mobility in mycolate membranes, we considered their self-diffusion coefficients.

### 2.3. MA Self-Diffusion in Bilayer Membranes

The self-diffusion coefficients *D* of the MA molecules in the membranes were estimated from the analysis of the Mean Squared Displacement (MSD) of the instantaneous positions of their centers of mass from the initial positions (see Section 3.3). Several approaches aiming to ensure reliable *D* determination from the molecular dynamics trajectories were evaluated. At elevated temperatures (350 K and higher), it was found that all methods gave close *D* values, differing within 10–20% and usually falling within the calculated uncertainty intervals. At temperatures of 300–310 K, the *D* values differed significantly, sometimes exceeding the calculated uncertainty intervals. This fact, apparently, is explained by the high viscosity and inhomogeneity of membranes at low temperatures, which leads to the non-ideality of diffusion, its dependence on the local environment of the molecule and, finally, the strong nonlinearity of MSD dependence on time.

The self-diffusion coefficients of the AMA molecules in different membranes are given in Table 1, including the values of the one-dimensional diffusion coefficient *D_x_* in the OX direction, the lateral diffusion coefficient *D_xy_* in the OXY plane, and the total diffusion coefficient *D*. Other terms of the diffusion tensor can be found from the following relations:

$$D=\frac{1}{3}\left(D_{x}+D_{y}+D_{z}\right),\;D_{\alpha \beta }=\frac{1}{2}\left(D_{\alpha }+D_{\beta }\right),\;\alpha ,\beta =x,y,z$$

**Figure 4:**
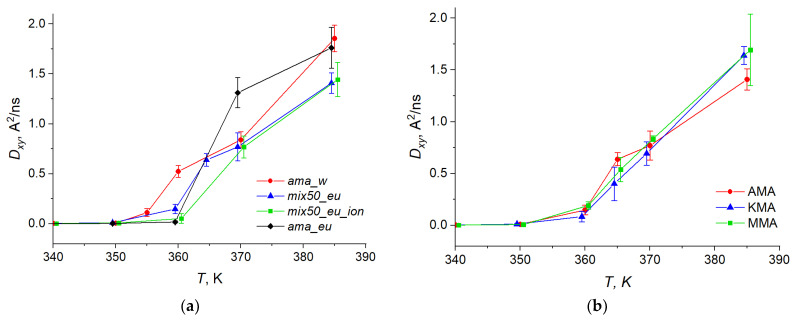
Temperature dependence of the lateral diffusion coefficients *D_xy_* for (**a**) AMA in various membranes; (**b**) AMA, KMA, MMA components in Mix50_eU. Points are slightly shifted in horizontal direction to avoid overlapping.

In the ionized membrane, the ionized and non-ionized AMA molecules were characterized by a common diffusion coefficient. Table 2 summarizes the diffusion coefficients of the KMA and MMA molecules in the Mix50_eU membrane. The temperature dependencies of the lateral diffusion coefficient *D_xy_* for different MA components in the membranes of different types are shown in Figure 4.

Based on these results, several conclusions can be drawn. The lateral self-diffusion coefficients of individual components in the Mix50_eU membrane differ slightly from each other, although in many cases these differences fall within the uncertainty intervals δ*D*. For the membranes of different types, the differences in the diffusion coefficients are more complex. Figure 4 shows that, at the temperatures of 355–370 K, the diffusion coefficients of the AMA molecules are slightly higher in the AMA_W and AMA_eU membranes, i.e., at higher temperatures, the AMA molecules in single-component membranes become noticeably more mobile. This provides additional confirmation of the conclusion made earlier [44], which is that, in the mixed membranes, the KMA and MMA molecules act as stabilizers of the membrane structure; this is also the case at elevated temperatures, because their oxygen-containing groups form additional hydrogen bonds with the aqueous phase, reducing the mobility of these and neighboring molecules within the membrane.

In the temperature range of 350–375 K, AMA_W is characterized by higher *D* values than AMA_eU. This can be explained by the fact that in this region, the more mobile AMA_W membrane undergoes a phase transition to the liquid crystalline state (see Section 2.4), while in AMA_eU, this transition occurs in the range of 360–370 K, where its diffusion coefficient becomes higher than in AMA_W. However, as soon as both membranes enter the liquid state and the transient processes are completed, the AMA_W diffusion coefficient again becomes slightly higher. In the low-temperature region (300–340 K), the diffusion coefficients of both membranes are small and, taking into account the confidence intervals, practically do not differ from each other.

**Figure 5:**
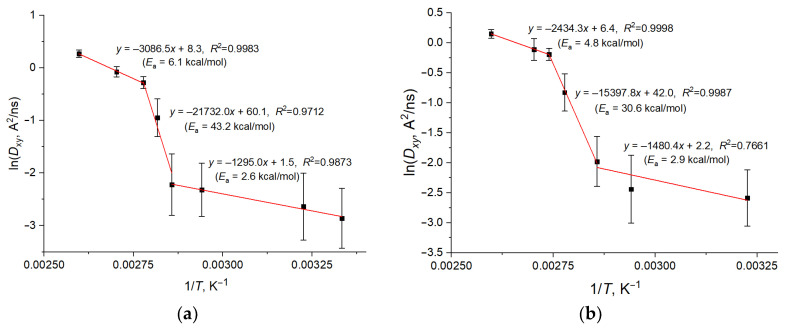
Dependence of ln(*D_xy_*) on the inverse temperature for (**a**) AMA in AMA_W; (**b**) AMA in Mix50_eU. The expressions on the panels give the linear regression models and the corresponding determination coefficients for three intervals of linear dependence. Values in parentheses are the apparent activation energies derived from the slope coefficients of linear intervals.

In the case of ideal diffusion, the temperature dependence of the diffusion coefficients *D* should obey the exponential law:

*D* = *D*_0_ exp(−*E*_a_/*RT*) (2)

where *D*_0_ is the preexponential factor; *R* is the universal gas constant; *T* is the absolute temperature, and *E*_a_ is the apparent activation energy of the process. This trend is also observed for the studied membranes, albeit only in certain temperature intervals. The dependencies of ln(*D_xy_*) on the inverse temperature are shown in Figure 5 for the AMA diffusion in the AMA_W and Mix50_eU membranes. The uncertainty intervals shown in the figure are calculated by the formula δ(ln*D*) = δ*D*/*D*. It can be seen that there are three distinctly different ranges of ln(*D_xy_*) linearity, and its determination errors are not consistent with a single linear dependence over the entire studied temperature range. This is due to the fact that a major rearrangement of the membrane structure (from gel to liquid crystal and the liquid melt, see Section 2.4) occurs in the region *T* = 355–370 K, which manifests as an increase in the diffusion coefficient. In the low-temperature linear section, the diffusion activation energy *E_a_* for the AMA_W membrane can be estimated as 2.6 kcal/mol, which is quite similar to the value obtained in the high-temperature section (6.1 kcal/mol). However, in the 355–370 K region, the diffusion coefficient increases strongly with increasing temperature, leading to an apparent value of *E_a_* = 43.2 kcal/mol. This sharp difference indicates a significant structural reorganization of the membrane over this temperature range. Comparable dependencies are found for other membranes, for example, Mix50_eU, where the apparent activation energies are about 3–5 kcal/mol in the low- and high-temperature sections and 30.6 kcal/mol in the medium section (Figure 5b). A similar pattern of changes in the diffusion coefficients in the phase transition region (separation of the ln*D* vs. 1/*T* dependence into several linear regions with different slope angles) was found in a study of supported lipid bilayers based on the coarse-grained MD modeling of five different lipids with different molecular architectures [54].

### 2.4. Temperature-Induced Structural Reordering in MA Membranes

The structural rearrangement evidenced by the nonlinearity of the temperature dependence of the diffusion coefficients is also visually apparent when analyzing the molecular structure of the membranes. With increasing temperature, the membrane changes from the fully ordered “gel” (L_o_) bilayer phase (characterized by the consistent conformations and orientations of the molecules), to the partially disordered liquid crystal bilayer state, and finally to the bulk liquid melt (3D disordered) phase.

In order to quantitatively describe the structural changes observed in membranes with increasing temperature, we considered the vectors representing the directions of individual chain fragments of MA molecules. For this purpose, the vectors between the functional group positions $r_{a},\;r_{b},\;r_{c},\;r_{d},\;r_{e}\;$in each molecule (points *a*, *b*, *c*, *d*, *e* in Figure 1), as well as the instantaneous position of the molecule’s center of mass $r_{0}$, were analyzed in each section of the MD trajectory. Based on the coordinates of these points, the vectors characterizing the directions of the chain fragments of AMA molecules can be determined (even though the chains themselves are not always linear):

$$r_{\alpha \beta }=r_{\beta }-r_{\alpha },\;\;\;\alpha ,\beta =0,a,b,c,d,e$$

**Figure 6:**
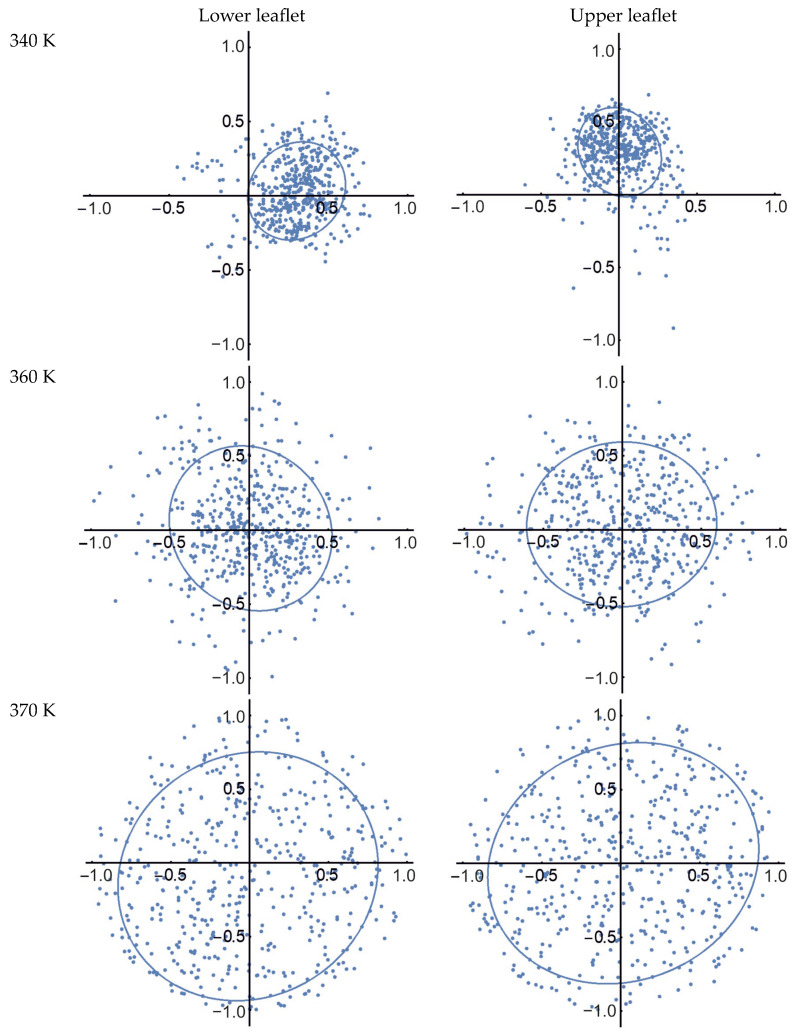
Changes in orientation and ordering of the AMA molecules in the Mix50_eU membrane (50 molecules per leaflet) over the 910–1000 ns trajectory section (10 frames at 10 ns steps) during heating from 340 K to 370 K. Each point represents the normalized vector $s_{i}$ projection on the OXY plane for each AMA molecule in a lower or upper membrane leaflet. Blue ellipses are the 3rd quartile ellipsoids for the point distributions. Coordinate axes correspond to the OX and OY directions in the membrane plane; values on axes are in the ranges of 0–1 (dimensionless).

Special attention was paid to the vector $r_{0b}$, which corresponds to the direction from the center of mass of the molecule to the COOH group (CM → COOH). This vector characterizes the “general direction” of the molecule in the membrane, usually pointing from the center of the membrane to its surface. The amount and direction of the molecule’s tilt relative to the membrane plane can be characterized by the projection of the normalized vector $r_{\alpha \beta }$ onto the OXY plane (which is parallel to the membrane plane in the simulation cell), i.e., the two-dimensional vector s:

$$s=\frac{\left(x_{0b},y_{0b}\right)}{\sqrt{x_{0b}^{2}+y_{0b}^{2}}}$$

where *x* and *y* are the components of the vector $r_{0b}$. Thus, the orientation of each molecule *i* in the membrane is represented by a two-dimensional normalized vector $s_{i}$, which, at any moment in time, falls within a unit circle in the OXY plane. The distribution of vectors $s_{i}$ characterizes the directional ordering of MA molecules in the membrane. Figure 6 shows the distributions of the orientation vectors $s_{i}$ of the AMA molecules in the Mix50_eU membrane at three different temperatures (see also Table S1 in the Supplementary Materials for the time evolution of $s_{i}$ in the same membrane at *T* = 340 K). The left and right panels correspond to the lower and upper membrane leaflets. The plots also show the (third) quartile ellipsoids, which cover three quarters of the distributed points.

It can easily be seen that at 340 K, there is a preferential cross-tilt of molecules, with different directions in the lower and upper leaflets (the preferential tilt directions differ by about 90°). When the temperature is increased to 360 K, the tilt pattern of the molecules substantially changes and the preferential tilt directions disappear in both leaflets. In addition, the tilt variance becomes much more pronounced (the quartile ellipsoids cover a larger area, their centers are close to the coordinate origin, and their eccentricity decreases significantly). Finally, when the temperature reaches 370 K, the area of the ellipsoids is further increased, reflecting much stronger variance in the orientation of the molecules.

**Figure 7:**
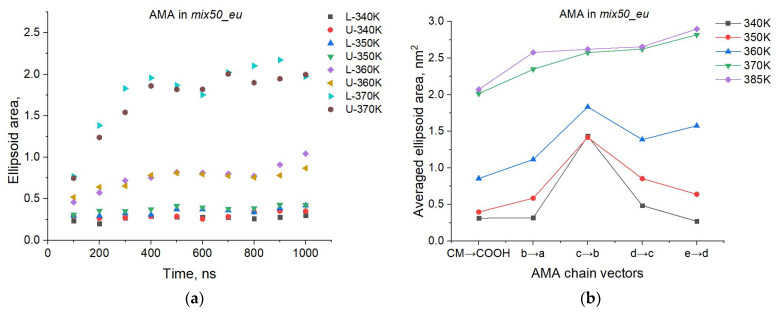
Trends and patterns in the orientation and ordering of MA molecules in the membranes. (**a**): Temporal changes in the 3rd quartile ellipsoid area *S* of the AMA orientation vectors (CM → COOH) depending on the temperature in the lower (L-) and upper (U-) leaflets of the Mix50_eU membrane. (**b**): Temperature-induced changes in area *S* for different AMA chain vectors (averaged over both membrane leaflets).

The size of quartile ellipsoids can be characterized by their area S=πab (*a*, *b* are the ellipsoid semiaxes), which varies with time and depends on the temperature. For this analysis, we used the averaged *S* values at different time points of the 0–1000 ns MD trajectories (in 100 ns steps) for different membranes at different temperatures. Figure 7a shows the typical changes in *S* for the AMA molecules in the top and bottom leaflets of the Mix50_eU membrane over time from 0 to 1000 ns with increasing temperature. It can be seen that there are no significant *S* changes with time at the temperatures of 340 and 350 K. This means that the tilt angles of the AMA molecules fluctuate within narrow limits and that the membrane remains structurally ordered. When the temperature increases to 360 K and especially to 370 K, a significant increase in *S* is observed, reflecting a strongly disordered orientation of the molecules.

These patterns and trends are observed in all the studied membranes. In addition, similar trends also occur for other $r_{\alpha \beta }$, i.e., for the orientation of other molecular sites. This is demonstrated in Figure 7b, which shows the changes in the area *S* of the quartile ellipsoids for the vectors $r_{0b},\;r_{ba},\;r_{cb},\;r_{dc},\;r_{ed}$. It can be seen that the increase in *S* with temperature has the same monotonic character for all sites of the MA molecules.

**Figure 8:**
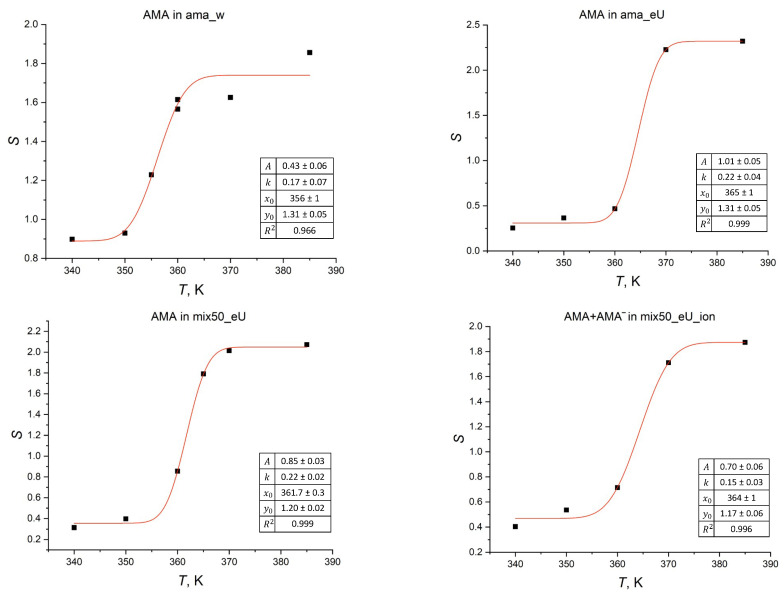
Temperature-induced changes in the ordering parameter *S* for AMA molecules in different membranes. Values in tables give the regression coefficients and statistics for the approximation model based on the erf(*x*) function (3). Individual data points (black squares) and regression model (red line) are shown.

In the later parts of the MD trajectories (*t* > 600 ns), the changes in *S* become insignificant. This indicates that the membranes are close to the equilibrated state. Thus, the *S* values in this time range characterize the membrane as a whole at a given temperature. To compare these states, the value of *S*_avg_ was used, which is the average value of *S* for the three moments in time (800, 900, 1000 ns). Figure 8 shows the dependence of *S*_avg_ (black squares) on the temperature for different membranes. It can be easily seen that in all membranes, it has a step-like form that corresponds to structural phase transitions around 350–360 or 360–370 K. This dependence can be approximated by a regression model based on the error function erf(*x*):

*y* = *A* · erf(*k*(*x* − *x*_0_)) + *y*_0_ (3)

The optimized regression parameters and the statistical characteristics of the models are shown in Figure 8. As can be seen, the phase transition temperature (represented by the *x*_0_ parameter) for the AMA_W membranes is 355 K, while for the membranes based on the eU conformations, this temperature is noticeably higher, ranging from 361 to 365 K. This difference is consistent with the conclusions made in the previous studies, which are that the membranes based on the elongated eU conformations are significantly more stable than the others. Moreover, the transition temperature is very weakly influenced by the degree of ionization of the membrane molecules (i.e., pH of the medium) or the presence of the KMA and MMA components.

The temperature interval in which a jump in the ordering parameter *S* occurs is consistent with the interval in which there is a spike-like increase in the diffusion coefficient (Figure 5). The observed jumps in these parameters can be considered as a phase transition of the first kind of the order–disorder type, which corresponds to the transition from a gel-like to a more mobile, partially disordered liquid crystalline state and then to the fully disordered liquid melt state [55]. It can be seen that such a transition occurs in a rather wide temperature range, which is usually characterized as a “blurred” phase transition.

The time duration over which the phase transition occurs can be estimated from the initial sections of the time-dependence curves in Figure 7a. Since the temperature setting time for the used MD thermostat is small (usually within fractions of a nanosecond), the steep section of the trajectory corresponds to the changes in the system state induced by the thermostat temperature. In this model system, the transition duration is 400–800 ns.

**Figure 9:**
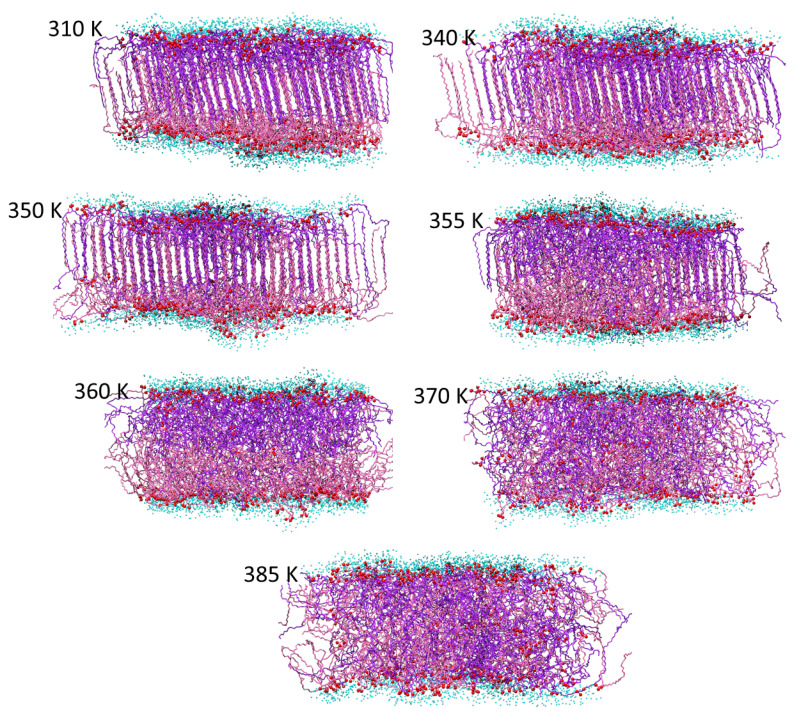
Final structures of the AMA_W membrane at different temperatures. The AMA hydrocarbon chains are represented by purple stick models (darker and lighter color shades correspond to the molecules originally located in the upper and lower membrane leaflets, respectively). Red balls represent MA oxygen atoms and small cyan balls represent oxygen atoms of water molecules within 5 Å from membrane.

**Figure 10:**
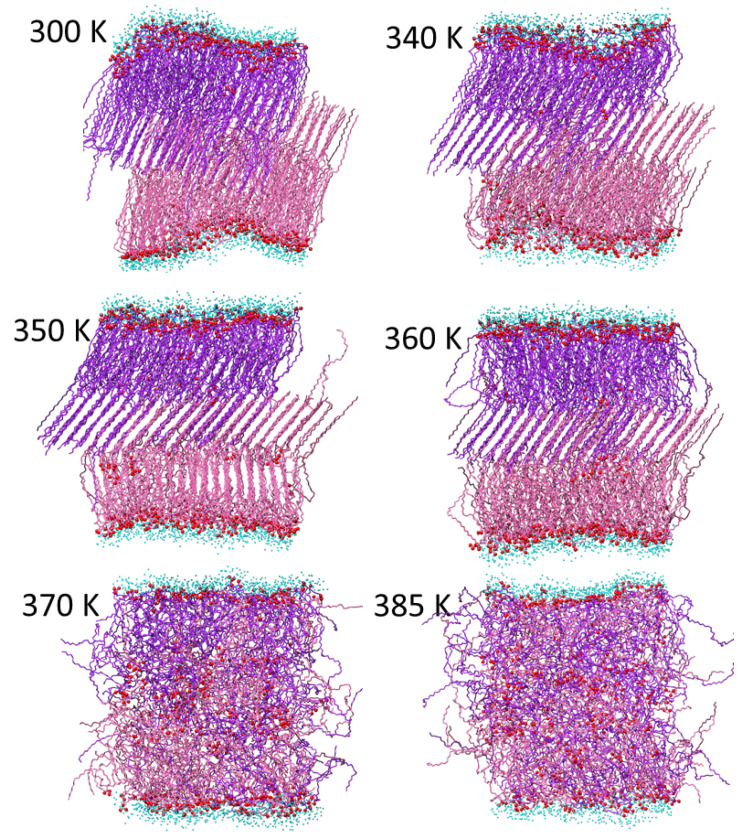
Final structures of the AMA_eU membrane at different temperatures. The AMA hydrocarbon chains are represented by purple stick models (darker and lighter color shades correspond to the molecules originally located in the upper and lower membrane leaflets, respectively). Red balls represent MA oxygen atoms and small cyan balls represent oxygen atoms of water molecules within 5 Å from membrane.

**Figure 11:**
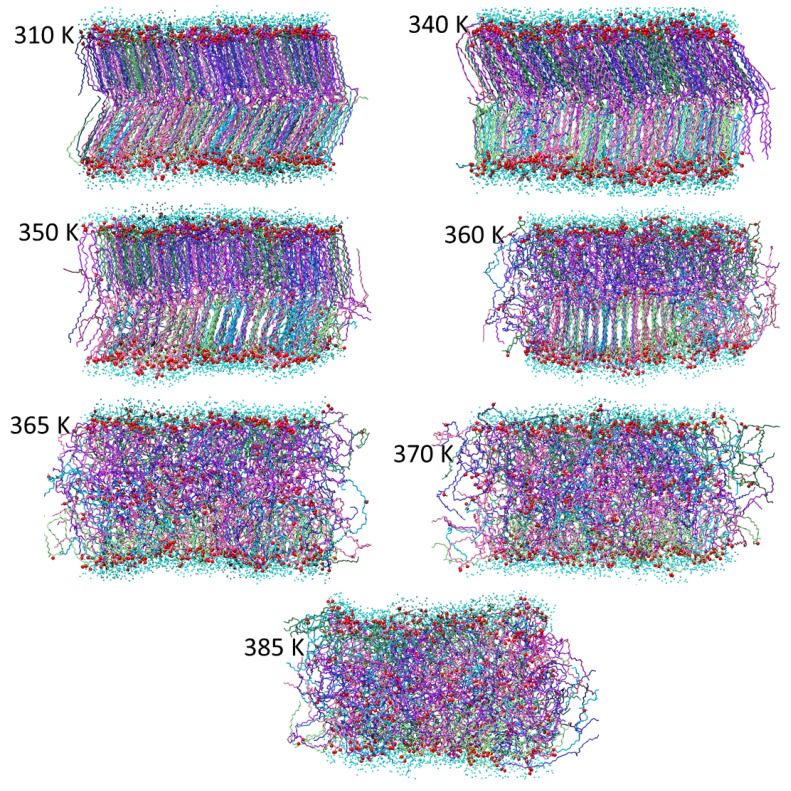
Final structures of the Mix50_eU membrane at different temperatures. The AMA, MMA, and KMA hydrocarbon chains are represented by purple, blue, and green stick models (darker and lighter color shades correspond to the molecules originally located in the upper and lower membrane leaflets, respectively). Red balls represent MA oxygen atoms and small cyan balls represent oxygen atoms of water molecules within 5 Å from membrane.

The conclusions regarding the membrane phase transitions are further confirmed by the direct inspection of the final (end of trajectory) membrane structures at the different temperatures shown in Figure 9, Figure 10 and Figure 11 for the AMA_W, AMA_eU, and Mix50_eU membranes, respectively. At low temperatures (300–310 K), the gel-like structures with polar groups exposed to the water are observed. In AMA_W, the MA molecules of both leaflets are tilted in a similar way and fully interdigitated. In AMA_eU, three layers cross-tilted at different angles are observed, with the middle layer formed by interdigitated tails of the elongated meromycolate chains. In Mix50_eU, there are two cross-tilted layers linked by interdigitated tails of the elongated AMA meromycolate chains. At a moderate temperature (340 K), the membranes retain these basic structures; however, in some cases, the orientation of the molecules may be slightly changed and the membranes may become slightly more ordered with respect to slant angles and interface surfaces.

At 350–355 K, the AMA_W membrane transitions into a partially disordered liquid crystal phase and then into a partially melted one while a certain degree of ordering is retained in some areas. At 360 K, the orientational ordering is lost, but the transverse localization of MA molecules and polar groups is partially retained. Finally, at 370 K and above, a fully disordered melted liquid phase with random positions and conformations of the molecules is formed.

In AMA_eU, the phase transition also starts at about 350 K but requires higher temperatures. At 350–360 K, the membrane retains some order, especially in the middle layer, while melting starts in the lower and upper layers. At 370 K, some transverse localization is still present and the fully disordered melted phase is formed closer to 385 K.

Finally, in Mix50_eU, partial and then full melting is observed from 350 to 365 K in one and then in both layers, and a fully disordered liquid phase is formed at 370 K and above.

It should be noted that the phase transition temperatures determined from the MD simulations appear to be overestimated by 20–30 K compared to the melting temperatures of solid mycolic acids. In addition, as discussed in the Introduction section, in most membranes extracted from various microorganisms, the experimentally recorded phase transitions are observed in the temperature range of 330–340 K, much lower than found in the present work. However, the structure and thermodynamics of the bulk preparations of solid MAs or cell wall components might not be directly translatable to the bilayer membranes. On the other hand, the pure mycolic acid bilayer membranes are clearly not a perfect model of the natural mycobacterial membrane. Even during the early studies of Mtb membranes, a model was proposed [56] wherein the mycolic acid molecules covalently bound to the underlying arabinogalactan layer of the cell wall provided a dense and weakly mobile base for the formation of the upper leaflet containing a variety of glycolipids and having much higher fluidity than the mycolate layer. It is possible that the high fluidity of the outer membrane observed in the experiments is due to this more mobile layer. In addition, the higher phase transition temperatures may also be caused by the calibration deficiencies of the force field used, which was developed to describe biological systems in the typical biochemical temperature ranges without careful adjustment to the properties of systems at elevated temperatures. This is indirectly confirmed by the fact that in the water model used (SPC216), the boiling point is about 120 °C.

It should also be taken into account that, for some microorganisms (e.g., *M. terrae*), the membrane phase transitions are observed in the region of 90–95 °C. This may indicate that a certain change in the component composition or packing of mycolic acid molecules in these membranes gives rise to a significantly higher thermal stability. Generally, microorganisms respond to heat stress via massive changes in gene expression that, among others, adjust the membrane and cell wall compositions in order to maintain the required levels of fluidity, permeability, and biosynthesis [57,58]. Unfortunately, no systematic, sufficiently detailed and precise lipidome studies for the heat-resistant mycobacteria species or strains are currently available. The observed effects for *M. thermoresistibile* involve a dramatic decrease in the synthesis of cyclopropanated AMA, MMA, and KMA, as well a decrease in the synthesis of trehalose dimycolates and in the expression of enzymes involved in the final stages of mycolic acid synthesis and transfer [23,59]. It is suggested that the concomitant accumulation of their *cis*-olefin precursors (including α’-mycolates that have a shorter meromycolate chain with a double bond) may represent a strategy able to increase the cell wall fluidity [23]. However, in other species, the high-temperature adaptation patterns may be substantially different [57], and the overall picture is not yet clear. In this light, the structural organization and component composition of the membranes studied in this work might correspond to the thermostable membrane varieties of real organisms. Nevertheless, regardless of the reasons for the overestimation of the absolute phase transition temperatures, the relative changes in the structural features of the membranes and the characteristic times of the changes induced by an increase in temperature should not depend significantly on the absolute temperature values at which the phase transitions occur in the model systems. This also applies to the comparisons of features between individual membranes and between the individual components of a single membrane established here.

Thus, the results obtained in this work show that the mobility and fluidity of the pure mycolic membranes cannot explain the high fluidity of the outer membrane of mycobacteria, or at least all of their representatives. To properly model the outer membrane, more complex membrane compositions should be used, possibly saturated with the lighter lipids or glycolipids that are present in the outer leaflet of the outer membrane of Mtb or in the capsule layer around it.

## 3. Materials and Methods

### 3.1. Model Membranes

As explained in Section 2.1, several symmetrical bilayer membranes formed by free MA molecules were considered in this study. Three of them were adopted from our previous study (refer to publication [44] for the method of their construction, optimization and equilibration, as well as for a detailed description of their conformational composition and its evolution over time). The first one, designated as AMA_W, was initially constructed from the AMA molecules in their W conformations (see [44] for the conformation designations) that undergo significant conformational restructuring. The second pure AMA bilayer membrane, designated as AMA_eU and built with the initial eU conformations, had the greatest density and thickness. The third was a mixed membrane constructed from three components: AMA (50%), KMA (25%), and MMA (25%), with the AMA molecules initially having eU conformations. This composition was close to the natural composition of the Mtb outer membrane and was designated as Mix50_eU. Finally, in order to estimate the influence of the environmental acidity, its partially ionized form Mix50_eU_ion was additionally considered. It is known that, at pH = 4.5 (typical pH in the internal volume of phagolysosomes, where Mtb resides in the latent form of TB), about 20% of mycolic acid molecules are ionized. Thus, in the ionized membrane, 20 randomly selected MA molecules of the Mix50_eU membrane were taken in their ionized state R—COO^−^.

The chemical and conformational compositions of the studied membranes, as well as their structure and density characteristics, are presented in detail in Table 3. The thickness and density of the membranes correspond to the state after NPT equilibration for a duration of 300 ns at 300 K in aqueous media. The dimensions of the simulation box and the details of the MD simulations are described in Section 3.2. In the neutral systems, no ions were added for simplicity (similar to the approach taken, e.g., in [60,61]), since the influence of the solution ionic strength on the MA polar groups and the temperature-dependent membrane behavior was expected to be insignificant. For the ionized Mix50_eU_ion membrane, additional 40 Na^+^ ions were added to the box in order to neutralize the charges; this was followed by the MD equilibration (see Section 3.2).

### 3.2. Molecular Dynamics Simulations

In all MD simulations, the all-atom CHARMM36 force field [62] was used, which belongs to the modern family of force fields, continues to be actively improved, and is recommended for modeling lipid membranes [63]. The MA topologies were obtained using the CHARMM-GUI automatic topology builder [64,65]. For the ionized Mix50_eU_ion membrane, additional 40 Na^+^ ions were added to the system in order to neutralize the charges. The number of atoms in the simulated system comprising the membrane, water (water model SPC216), and ions (if necessary) was 140–160 thousand. The simulation was carried out by using the GROMACS 2022.2 software package (GROMACS development team, https://www.gromacs.org/, accessed on 4 December 2023) [66] at the 24 core workstations with the NVIDIA GeForce RTX 3080Ti GPU.

Equilibration of the ionized Mix50_eU_ion membrane was performed in several stages: (1) energy minimization (up to *F_max_* ≤ 1000 kJ/mol); (2) NPT ensemble simulation with the “frozen” MA atoms (*t* = 100 ps, v-rescale thermostat, Parrinello-Rahman barostat, *T* = 310 K, *P* = 1 bar); and (3) NPT ensemble simulation with “unfrozen” MA atoms (*t* = 100 ns, v-rescale thermostat, Parrinello-Rahman barostat, *T* = 310 K, *P* = 1 bar). Typically, a semi-isotropic barostat is recommended for use in membrane calculations [67]. However, it was demonstrated recently [68] that the diffusion constants estimated with this method do not converge to the proper macroscopic values, which is critical for the purposes of our study. Therefore, an isotropic barostat was used in the course of this work.

After the initial equilibration at 300 K, the production NPT runs were performed at constant temperatures in the ranges of 310–385 K. The point 385 K was added to ensure the proper asymptotic behavior of the structure disorder parameters at high temperatures. Because the SPC water boils at about 390 K, it enables the overheated water to be modelled. The duration of the production runs was 1000 ns, and the final part of the trajectory of 600–1000 ns was considered during the restructuring analysis.

For the analysis and visualization of the results, the USCF Chimera 1.15 software was used (University of California San Francisco, San Francisco, CA, USA, https://www.cgl.ucsf.edu/chimera/, accessed on 4 December 2023) [69].

For the analysis of the MA molecule orientation, the quartile ellipsoids of the bivariate distributions were calculated using a Python script.

### 3.3. MA Self-Diffusion in Bilayer Membranes

The self-diffusion coefficients of the MA molecules in the membranes can be estimated from the analysis of the Mean Squared Displacement (MSD) of the instantaneous positions of their centers of mass $r\left(t\right)$ from the initial positions $r\left(0\right),$ as follows:

(4) $$MSD\left(t\right)=\langle \;{\left|r\left(t\right)-r(0)\right|}^{2}\;\rangle$$

where the angle brackets denote averaging over all the considered molecules in the membrane. In the case of the ideal Brownian diffusion of free particles in a weakly viscous medium, MSD is linearly dependent on time, with the diffusion coefficient defined by the following equation:

(5) $$D=\frac{1}{2d}\frac{\partial MSD\left(t\right)}{\partial t}$$

where *d* denotes the number of space dimensions in which diffusion is considered. In a nonideal medium, the dependence $MSD\left(t\right)$ in most cases is far from linear and the diffusion coefficients cannot be determined with good accuracy. Recently, a large number of studies have appeared suggesting ways to improve the accuracy of *D* determination in the case of non-ideal diffusion [70,71,72]. In addition, it has recently been shown that the calculation of *D* under NPT dynamics conditions is particularly challenging due to the need to properly account for periodic conditions [73,74,75] and the fact that not all standard programs provide such accounting. In the course of the visual analysis of MD trajectories, we also found that some molecules make large jumps between periodic cells, which leads to a significant overestimation of *D*. These effects are especially noticeable at temperatures *T* > 350 K. Therefore, in this paper, we used three approaches to estimate *D*: (i) calculation using the “naïve” algorithm (4)–(5); (ii) calculation using the ordinary least squares estimator (OLS) described in [74], which additionally averages the MSD over time intervals with different lag times; and (iii) the method implemented in the VMD program (variants of this method are described in [76] and represent a modification of OLS). When calculating *D* by methods (i) and (ii), we used our in-house program that implements a slightly modified version of the TOR unwrapping procedure proposed in [75], with the additional visual control of particle trajectories to ensure the absence of unphysical particle jumps and the immobility of the center of mass of the system. Using method (ii), the uncertainty was estimated as the standard deviation from the mean value of the *D* coefficients calculated at four equal consecutive intervals of the trajectory under study.

It was found that at elevated temperatures (350 K and higher), all methods gave close *D* values, differing within 10–20% and usually falling within the calculated uncertainty intervals. At temperatures of 300–310 K, the *D* values differed significantly, sometimes exceeding the calculated uncertainty intervals. Taking this into account, method (ii) was further employed to estimate the self-diffusion coefficients of the MA molecules in the different membranes reported in this study.

## 4. Conclusions

In this study, we have shown that microsecond-scale all-atom molecular dynamics simulations can be employed to obtain meaningful results regarding the temperature-induced structural changes and thermal stability of bilayer mycolic acid membranes. The conformational drift of the MA membrane molecules in a wide temperature range (340–370 K) follows the first-order reaction kinetics with activation energies of about 6.1 kcal/mol. The characteristic times of conformation changes in the model membranes are 80–160 ns, in agreement with the previously obtained results [44].

The lateral self-diffusion coefficients of the MA molecules in the membranes vary from thousandths to single-digit Å^2^/ns values in the temperature range of 310–370 K. Regarding the temperature dependence of the diffusion coefficients, three sections are observed that correspond to the apparent diffusion activation energies of about 2–7 kcal/mol at low or high temperatures (*T* = 310–350 K or *T* > 370 K) and of about 30–40 kcal/mol in the intermediate temperature range.

In the single-component membranes, the diffusion coefficients depend, in a complex way, on the membrane packing; meanwhile, in the multi-component ones, the diffusion coefficients of individual components differ only slightly. The influence of the degree of ionization of the membrane molecules (i.e., external pH) on the diffusion coefficients is small.

At low temperatures (*T* < 340 K), a significant degree of orientational ordering (tilt) is observed in the MA molecules. When the temperature is increased, the membrane attains a partially disordered liquid crystal bilayer state and then reaches the bulk liquid melt (3D disordered) phase, corresponding to a “blurred” phase transition of the first kind of the order–disorder type. The phase transition temperatures in the membranes with initial W conformational packing are about 10 K lower than in the membranes with the eU packing. At the same time, the differences in the transition temperatures for the single-component and multi-component membranes based on the eU packing are much less pronounced. The characteristic times of phase transitions in the model membranes are 400–800 ns.

Overall, a better understanding of the structure and behavior of mycobacterial membranes could be useful for the prevention of drug resistance and the analysis of factors influencing the heat resistance of mycobacteria, as well as in the development of membrane-targeting pharmaceuticals and the design of novel membrane-based materials.

## Figures and Tables

**Table 1:** Self-diffusion coefficients of AMA molecules in various membranes.

*T*, K	*D_x_*, Å^2^/ns	*D_xy_*, Å^2^/ns	*D*, Å^2^/ns
AMA in AMA_W			
300	0.001 ± 0.001	0.001 ± 0.001	0.003 ± 0.011
310	0.002 ± 0.002	0.002 ± 0.002	0.003 ± 0.003
340	0.004 ± 0.003	0.005 ± 0.002	0.006 ± 0.004
350	0.007 ± 0.003	0.006 ± 0.004	0.008 ± 0.006
355	0.131 ± 0.061	0.112 ± 0.040	0.086 ± 0.034
360	0.470 ± 0.083	0.524 ± 0.058	0.359 ± 0.044
370	0.802 ± 0.180	0.840 ± 0.082	0.601 ± 0.055
385	1.731 ± 0.375	1.856 ± 0.132	1.299 ± 0.087
AMA in AMA_eU			
310	0.005 ± 0.002	0.004 ± 0.002	0.005 ± 0.003
340	0.001 ± 0.001	0.001 ± 0.001	0.001 ± 0.001
350	0.004 ± 0.003	0.004 ± 0.002	0.003 ± 0.002
360	0.019 ± 0.012	0.018 ± 0.010	0.013 ± 0.007
370	1.329 ± 0.163	1.312 ± 0.151	0.998 ± 0.129
385	1.822 ± 0.235	1.761 ± 0.204	1.366 ± 0.166
AMA in Mix50_eU			
310	0.002 ± 0.002	0.003 ± 0.001	0.003 ± 0.002
340	0.003 ± 0.002	0.004 ± 0.002	0.003 ± 0.002
350	0.010 ± 0.005	0.010 ± 0.004	0.008 ± 0.004
360	0.129 ± 0.042	0.148 ± 0.046	0.106 ± 0.036
365	0.598 ± 0.051	0.638 ± 0.063	0.478 ± 0.047
370	0.738 ± 0.179	0.770 ± 0.140	0.589 ± 0.113
385	1.442 ± 0.120	1.408 ± 0.102	1.065 ± 0.100
(AMA + AMA^−^) in Mix50_eU_ion			
310	0.001 ± 0.002	0.003 ± 0.001	0.003 ± 0.003
340	0.002 ± 0.001	0.002 ± 0.001	0.002 ± 0.001
350	0.010 ± 0.009	0.010 ± 0.009	0.007 ± 0.008
360	0.060 ± 0.027	0.054 ± 0.036	0.042 ± 0.029
370	0.635 ± 0.095	0.789 ± 0.108	0.575 ± 0.064
385	1.406 ± 0.261	1.526 ± 0.040	1.107 ± 0.050

**Table 2:** Diffusion coefficients of KMA and MMA molecules in the Mix50_eU membranes.

*T*, K	*D_x_*, Å^2^/ns	*D_xy_*, Å^2^/ns	*D*, Å^2^/ns
KMA in Mix50_eU			
310	0.002 ± 0.002	0.003 ± 0.001	0.003 ± 0.002
340	0.002 ± 0.002	0.002 ± 0.001	0.002 ± 0.001
350	0.011 ± 0.006	0.012 ± 0.003	0.009 ± 0.003
360	0.071 ± 0.038	0.084 ± 0.050	0.074 ± 0.032
365	0.323 ± 0.149	0.402 ± 0.161	0.314 ± 0.115
370	0.755 ± 0.246	0.694 ± 0.113	0.503 ± 0.076
385	1.358 ± 0.325	1.641 ± 0.087	1.195 ± 0.083
MMA in Mix50_eU			
310	0.003 ± 0.003	0.003 ± 0.001	0.003 ± 0.002
340	0.002 ± 0.003	0.003 ± 0.002	0.002 ± 0.002
350	0.005 ± 0.005	0.007 ± 0.003	0.006 ± 0.003
360	0.195 ± 0.036	0.192 ± 0.037	0.146 ± 0.033
365	0.476 ± 0.130	0.539 ± 0.118	0.385 ± 0.109
370	1.075 ± 0.085	0.837 ± 0.027	0.623 ± 0.035
385	1.897 ± 0.224	1.694 ± 0.344	1.274 ± 0.288

**Table 3:** Composition of model membranes and their properties at the initial state of *T* = 300 K.

Membrane	AMA_W	AMA_eU	Mix50_eU	Mix50_eU_ion
Number of MA molecules in a box AMA:KMA:MMA	200:0:0	200:0:0	100:50:50	100:50:50
Initial conformations of AMA:KMA:MMA	W:–:–	eU:–:–	eU:W:W	eU:W:W
Number of ionized molecules AMA+KMA+MMA	0	0	0	20
Time of equilibration, ns	1200	300	300	100 ^a^
Thickness after equilibration, nm	4.4	7.8	5.0	5.1
Density after equilibration, kg/m^3^	863	907	877	873

Note: ^a^ Started from the Mix50_eU structure equilibrated for 300 ns.

## Data Availability

Data are contained within the article and Supplementary Materials.

## Supplementary Materials

The following supporting information can be downloaded at: https://www.mdpi.com/article/10.3390/molecules29030696/s1. Table S1. Temporal evolution of orientation vectors of AMA molecules in the upper and lower leaflets of the Mix50_eU membrane at *T* = 340 K.
