# Supplementary material for: Temperature-Induced Restructuring of Mycolic Acid Bilayers Modeling the Mycobacterium tuberculosis Outer Membrane: A Molecular Dynamics Study
Source: Molecules. 2024 Feb 2;29(3):696. doi: 10.3390/molecules29030696 (PMC10856651; doi:10.3390/molecules29030696)
Supplement: Supplementary file 1 [file molecules-29-00696-s001.zip › molecules-2831265-supplementary.pdf]

molecules

MDPI

Article

Temperature-Induced Restructuring of Mycolic Acid Bilayers Modeling the *Mycobacterium tuberculosis* Outer Membrane: A Molecular Dynamics Study

Alexander V. Vasyankin [1], Sergey V. Panteleev [1], Ilya S. Steshin [1], Ekaterina A. Shirokova [1], Alexey V. Rozhkov [1], Grigory D. Livshits [1], Eugene V. Radchenko [1,2], Stanislav K. Ignatov [1,*] and Vladimir A. Palyulin [1,2,*]

[1] Department of Chemistry, Lobachevsky State University of Nizhny Novgorod, Nizhny Novgorod 603022, Russia; alexandrvasyankin@gmail.com (A.V.V.), pasv1984@gmail.com (S.V.P.), ilya.steshin@icloud.com (I.S.S.), ekashirokova@gmail.com (E.A.S.), rozhkov@chem.unn.ru (A.V.R.), grigory.livshits@gmail.com (G.D.L.); genie@qsar.chem.msu.ru (E.V.R.)

[2] Department of Chemistry, Lomonosov Moscow State University, Moscow 119991, Russia

* Correspondence: ignatov@unn.ru (S.K.I.), vap@qsar.chem.msu.ru (V.A.P.)

Supplementary Materials

Table S1. Temporal evolution of orientation vectors of AMA molecules in the upper and lower leaflets of the Mix50_eU membrane at T = 340 K. Blue dots are the projections of the ends of the orientation vectors (CM→COOH) of individual molecules onto the OXY plane of the membrane. The horizontal axis is OX, the vertical axis is OY, and the coordinates on the axes are in nm. Blue ellipsoids represent the 3rd quartile spread of the vectors.

AMA in Mix50_eU at *T*=340 K		
t, ns	Lower leaflet	Upper leaflet
0–100	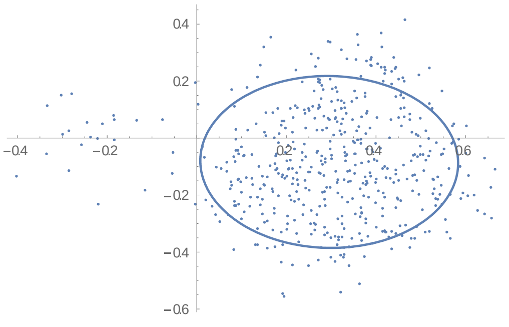	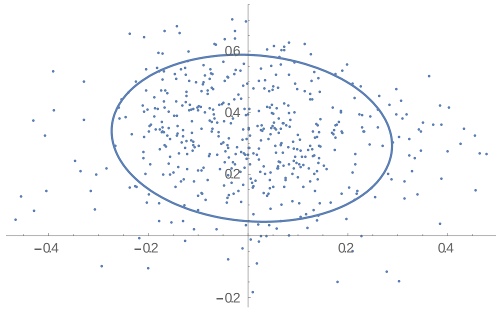
100–200	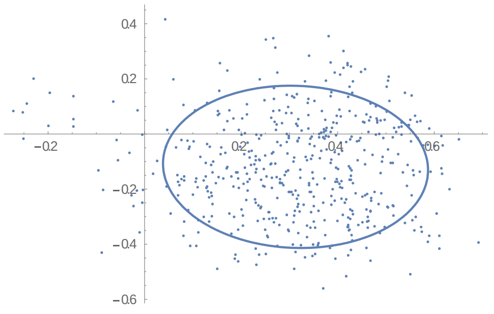	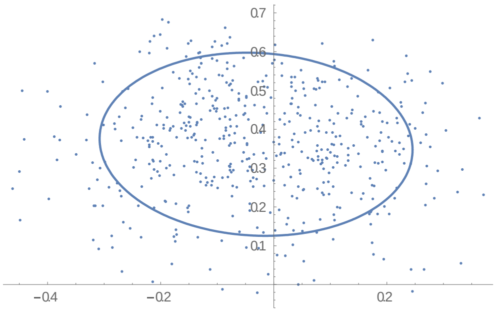

200–300	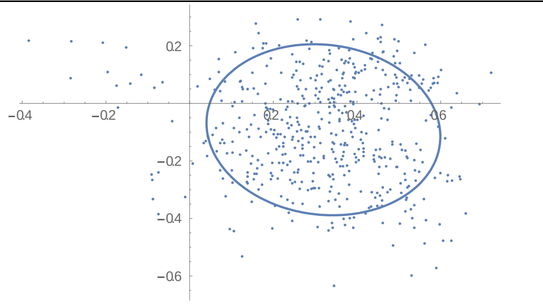	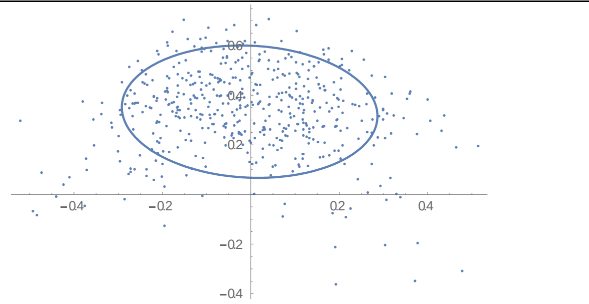
300–400	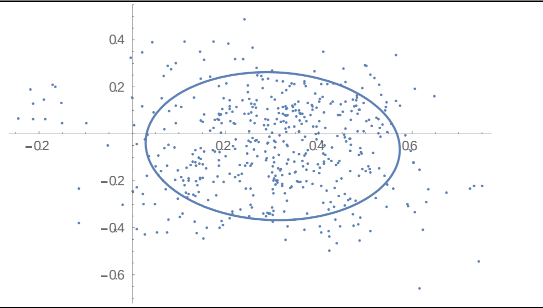	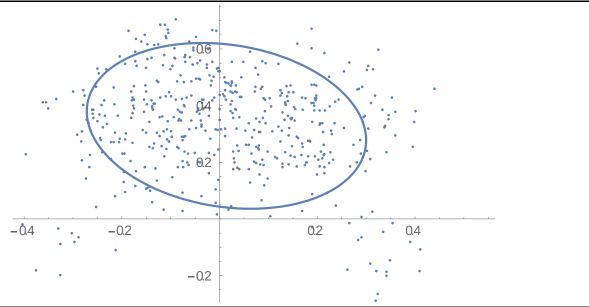
400–500	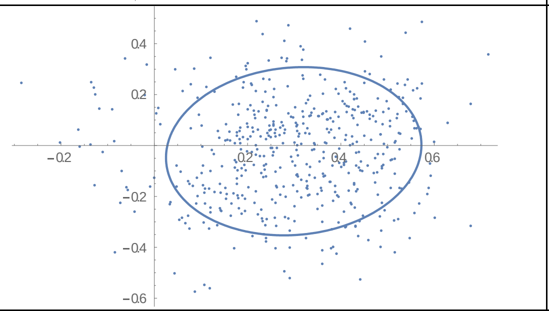	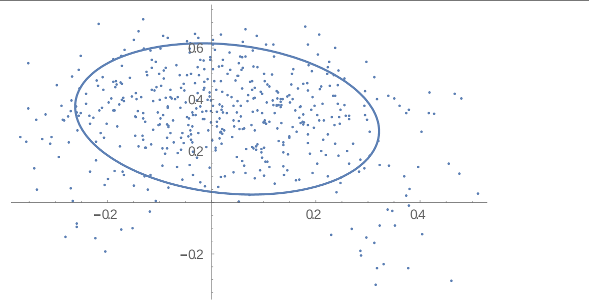
500–600	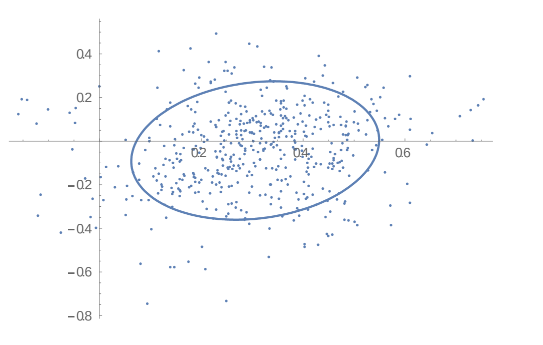	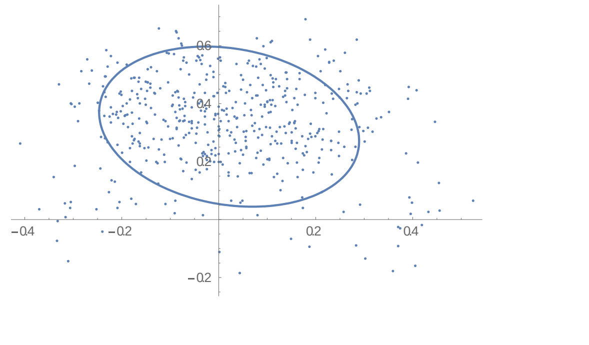
600–700	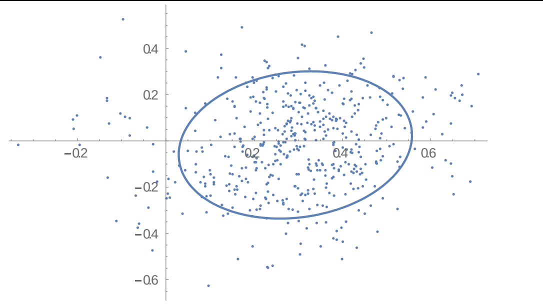	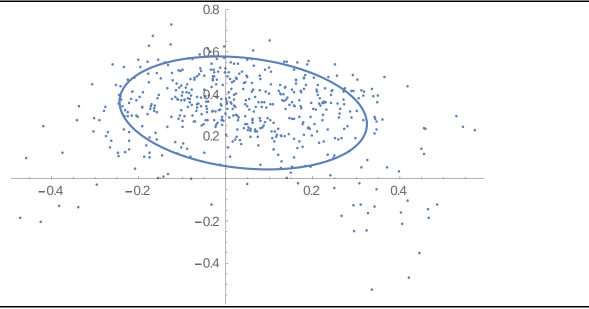

700–800	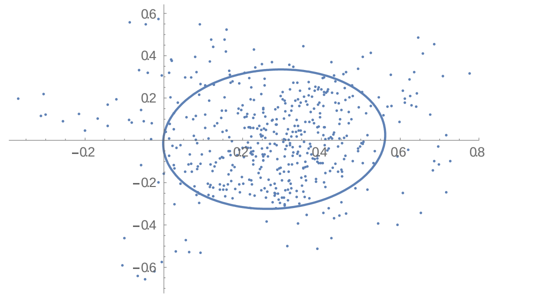	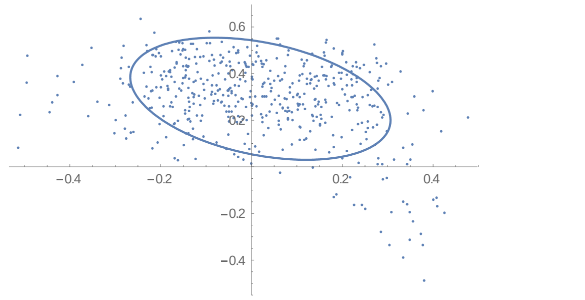
800–900	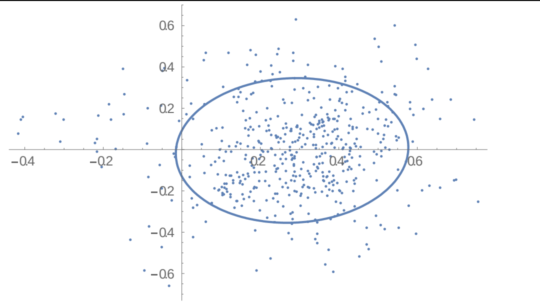	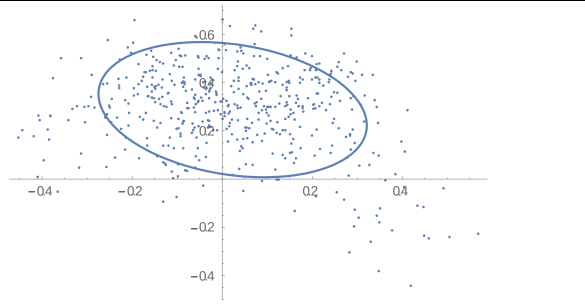
900–1000	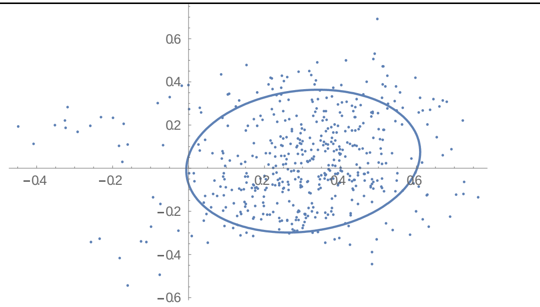	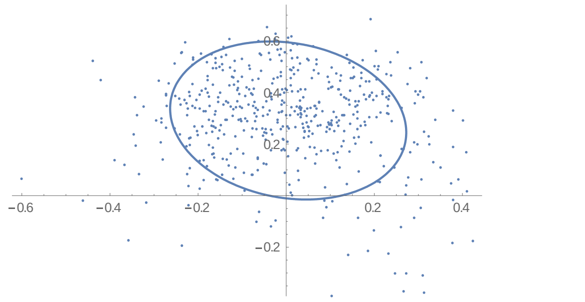
